# EGFR mutation: novel prognostic factor associated with immune infiltration in lower-grade glioma; an exploratory study

**DOI:** 10.1186/s12885-019-6384-8

**Published:** 2019-12-04

**Authors:** Zhaonian Hao, Dongsheng Guo

**Affiliations:** Department of Neurosurgery Tongji Hospital, Tongji Medical College, Huazhong University of Science and Technology, Wuhan, 430030 China

**Keywords:** Glioma, EGFR mutation, Immune infiltration, Immune microenvironment, PD-L1, PD-1 inhibitor

## Abstract

**Background:**

Glioma is one of the most common type of primary central nervous system tumors. EGFR mutation, a common alteration occurs in various tumors, is not brought to the forefront in understanding and treating glioma at present.

**Methods:**

In the present study, we demonstrated an immune infiltration related pattern of EGFR mutation in lower-grade glioma. In silico analyses were performed to investigate EGFR mutation and its biological effects and clinical values. GO and GSEA process were used as enrichment analysis. Infiltration levels of specific types of immune cells were estimated at TIMER database. Clinical data of patients were obtained from TCGA and were employed for survival analyses.

**Results:**

Here we revealed that EGFR mutation leads to an up-regulation of immune response related pathways and dismal prognosis in lower-grade glioma. Infiltration of CD4+ T cells, neutrophils, macrophages, and dendritic cells were significantly increased in EGFR-mutant cases. Infiltration of specific types of immune cells were correlated with shorter survival time. PD-L1 was elevated in EGFR-mutant cases and correlated with infiltration level of CD4+ T cells, neutrophils and dendritic cells.

**Conclusion:**

EGFR mutation indicates increasing infiltration of specific types of immune cells and poor prognosis in lower-grade glioma. Alteration of immune microenvironment since the EGFR mutation might influence the survival of glioma. We also provided a novel evidence and indicator of PD-1 inhibitor application in glioma.

## Background

Sequencing techniques have shown a huge potential on exploring the relationship between genome alterations and tumors [1]. Since the accomplishment of the Human Genome Project (HGP) and the launch of The Cancer Genome Atlas (TCGA), the accumulation of genetic knowledge on tumor is rocketing in recent decade. Pan-cancer analysis based on TCGA not merely revealed a panorama of alteration signatures of tumor-related genome, but also established the basis for comparative studies on relevant types of tumor [2].

Gliomas, the most common primary central nervous system (CNS) tumors, are classified as grade I to grade IV on the base of criteria established by the World Health Organization (WHO) [3] and characterized by a high recurrence frequency, high mortality rate and dismal prognosis [4, 5]. Several genes, including IDH1/2, TP53, PTEN and EGFR, were confirmed significantly recurrently mutated genes in glioma [6–10]. TP53 mutation is considered to be early event during the genesis of an astrocytoma, while the amplification of EGFR and loss of function or mutation of PTEN are features of higher grade glioma [11–13]. Based on the TCGA data, the most frequently mutated genes in lower-grade glioma (WHO grade II- grade III) were IDH1 (77.25%), TP53 (48.04%), ATRX (39.22%), CIC (22.75%), TTN (17.06%), PIK3CA (8.43%) and EGFR (6.86%), and the most prevalent mutations in glioblastoma (GBM) were PTEN (34.86%), TTN (32.57%), TP53 (31.55%), EGFR (26.97%), MUC16 (18.07%) and NF1 (12.98%). The difference in mutation frequency of EGFR in GBM and LGG aroused our interest.

EGFR, a member of receptor tyrosine kinase (RTKs) family, is playing a crucial role on regulating the intracellular signal pathways that affect cellular proliferation, cell survival, metabolism, invasion and metastasis etc. [14–16]. Previous studies have revealed several deletions and point mutations that were characterized by enhancing the activity of the EGFR. Among them, EGFRvIII was the most prevalent [14]. In contrast to the EGFR mutation residing in the intracellular kinase domain in lung cancer, gliomas harbor EGFR mutations in the extracellular domain, and this signature of EGFR alteration gave the explanation to the dismal effect on the treatment with Tyrosine kinase inhibitors (TKIs) such as erlotinib [17]. Further research proved that EGFRvIII mutation in glioma lead to activation of multiple RTKs, and these coactivation effects could initiate the downstream signaling cascade, resulting in erlotinib resistance [18]. Combined inhibition of multiple RTKs and immune check point inhibition thus became the potential novel strategy.

Infiltration of immune cells in tumor context is becoming one of the crucial indicator of prognosis in various cancers [19, 20], indicating the important influence of immune microenvironment on tumor prognosis. EGFR mutation was confirmed to play significant role in modifying tumor microenvironment in lung cancer. We hypothesize that EGFR mutation in glioma shares the similar characteristics in modifying immune microenvironment.

Therefore, we analyzed the RNA-sequencing data combined with clinical traits of patient from TCGA database to explore the immune related features of EGFR mutation in glioma, and a prospect on inferior overall survival in EGFR mutated gliomas was obtained.

## Methods

### Analysis of differentially expressed genes on EGFR-MUT LGGs vs EGFR-WT LGGs

**Fig. 1:**
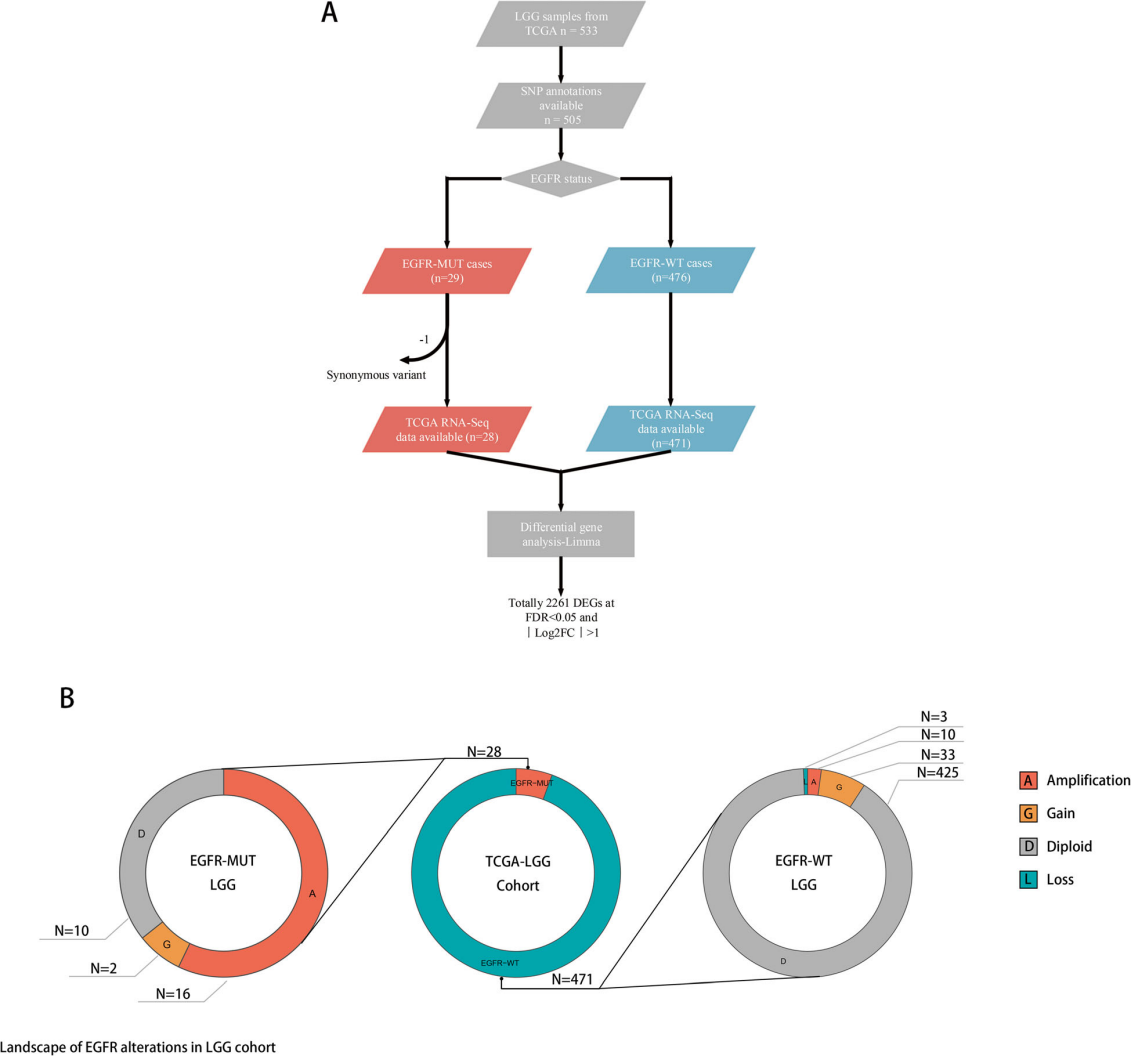
Flowchart of screening EGFR mutant LGG cases and landscape of EGFR alterations in LGG cohort. **a** LGG cases harboring EGFR-mutation were identified using MAF files from TCGA. 28 of 499 LGG cases were finally enrolled. All cases were verified with RNA-Seq, clinical data and SNP annotation available. Expression profiles of LGGs with EGFR mutation and corresponding wild-type control cases was analyzed using Limma in R to identify genes that were differentially expressed. **b** landscape of EGFR alterations in LGG cohort showed the population composition of the whole cohort

Data of 536 Lower-Grade glioma (LGG) cases (WHO grade II-III) were downloaded from TCGA in April 2019 at https://portal.gdc.cancer.gov/, including clinical data files, Mutation Annotation Format (MAF) files, RNA-seq files, HT-Seq files (files with number of reads aligning to each protein-coding gene), and FPKM-UQ files (files with number of fragments aligning per kilobase of transcript per million mapped reads normalized to upper quartile) were also acquired. The flowchart of selecting EGFR-MUT LGG cases was shown in Fig. 1.An EGFR mutation were identified using the MAF files. The somatic mutation variants data were extracted from TCGA files. Further annotation to reveal the biological significance of these variants were analyzed by using Variant Effect Predictor (VEP) at http://ensembl.org/info/docs/tools/vep/ website. Results shown in Additional file 1: Table S1. VEP prediction were presented as variants impact level and variation types, described as blow: variants with high impact indicating frame-shift or nonsense mutations, variants with moderate impact indicating missense mutations, and variants with low impact indicating synonymous mutations [21].

29 LGG cases with EGFR mutations were identified, one with synonymous mutation was excluded. 28 identified EGFR mutation were predicted to have high or moderate impact on EGFR function. Totally 499 LGG cases were eventually enrolled in the following analysis.

### Gene ontology (GO) analysis and gene set enrichment analysis (GSEA)

Differentially expressed genes of EGFR-MUT and EGFR-WT LGGs were generated using Limma package in R [22]. Enrichment analysis was performed at http://pantherdb.org/ to identify significant biological processes and pathways. The criteria of threshold were set at: FDR < 0.05, Log2FC > 1 or Log2FC < − 1 to generate gene list imported to PANTHER (version 14.1 released at April 2018) and the statistical significance was determined using Binomial test, and the Bonferroni correction was performed.

A pre-ranked GSEA analysis was performed using hallmark gene sets downloaded from Molecular Signature Database (http://software.broadinstitute.org/gsea/msigdb). Log2FC values of 19,753 transcripts generated from Limma by analyzing differentially expressed genes between EGFR-MUT and EGFR-WT LGGs were enrolled to create the pre-ranked gene list. Gene sets with FDR < 25% and specifically enriched at the beginning and end of the ranked list were considered to be enrichment significance [23, 24].

### Estimation of immune cells infiltration levels of EGFR-MUT and EGFR-WT cases

The infiltration levels of B cells, CD4+ T cells, CD8+ T cells, neutrophils, macrophages, and dendritic cells were obtained from Tumor IMmune Estimation Resource (TIMER) at https://cistrome.shinyapps.io/timer/ [25]. The data used to estimate infiltration levels were available at TCGA and transformed to Transcripts per million reads (TPM) format. Two-sided Wilcoxon rank test was used to determine the difference of immune cell infiltration between EGFR-MUT and EGFR-WT cases. Figures were plotted using R.

### Protein–protein interaction (PPI) network analysis

The protein–protein interaction (PPI) network of the identified survival associated hub genes was constructed using the STRING (Search Tool for the Retrieval of Interacting Genes) database at https://string-db.org/cgi/network.pl [26]. The PPI network was then visualized using Cytoscape software (version 3.6.0) [27].

### Survival analysis

Survival analysis was performed to investigate the difference on overall survival between EGFR-MUT and EGFR-WT cases, and also between subsets of relatively higher and lower infiltration level of immune cells. We then performed survival analysis to explore the effect of genes of interested on survival, which were picked up from the enrichment analysis of GSEA and GO. The Survival and Survminer packages were used to apply log-rank test (average values of factors were used as cutoffs) and the Kaplan-Meier curves were plotted.

COX proportional hazards models were established based on the gene sets generated from enriched pathways in EGFR-MUT or EGFR-WT cases. The COX proportional hazards test was performed in SPSS (version 19). Average values of covariates were used as cutoffs. The following log-rank test was performed using Survival and Survminer packages in R, and Kaplan-Meier curves were plotted in R.

## Results

### Immune response related genes are found to be up-regulated in EGFR-MUT LGGs

To explore the characteristics of EGFR mutations in lower grade glioma, we performed differential analysis of gene expression using RNA-seq data from TCGA-LGG cases with and without EGFR mutations. A total of 499 LGG cases, 471 of which had wild-type-EGFR (EGFR-WT) and 28 had mutant-EGFR (EGFR-MUT), were thus selected. And RNA-seq array data was available for all selected cases. In the TCGA cohort, the SNP frequency of EGFR is about 6.86%, and the rates of EGFR amplification and deletion in the whole cohort are 5.2% and 0.6% respectively (Fig. 1.B).

Raw data from EGFR-WT and EGFR-MUT cases, obtained from TCGA HT-seq files, were analyzed using Limma package of R for differential gene expression. 1598 genes were up-regulated in EGFR-MUT cases at FDR < 0.05(Log2FC > 1), while 663 genes were down-regulated in EGFR-MUT cases at the same threshold. EGFR was found to be up-regulated at a 2.3-fold (*p* = 1.69E-14) as well (Additional file 2: Table S2).

We further identified the biological processes enriched in the EGFR-MUT and EGFR-WT cases respectively, and enrichment analysis was employed among the differentially expressed genes using PANTHER. Genes involved in immune system process (*p* = 1.50E-12) and immune response (*p* = 4.93E-09) were enriched in EGFR-MUT LGGs, and simultaneously, genes participating in biological development were the top processes enriched in EGFR-MUT cases. Those genes related to nervous system development (*p* = 2.26E-22), neurogenesis (*p* = 4.50E-20) and generation of neurons (*p* = 1.30E-19) were top three processes enriched in EGFR-WT cases. Details shown in Additional file 3: Table S3.

**Fig. 2:**
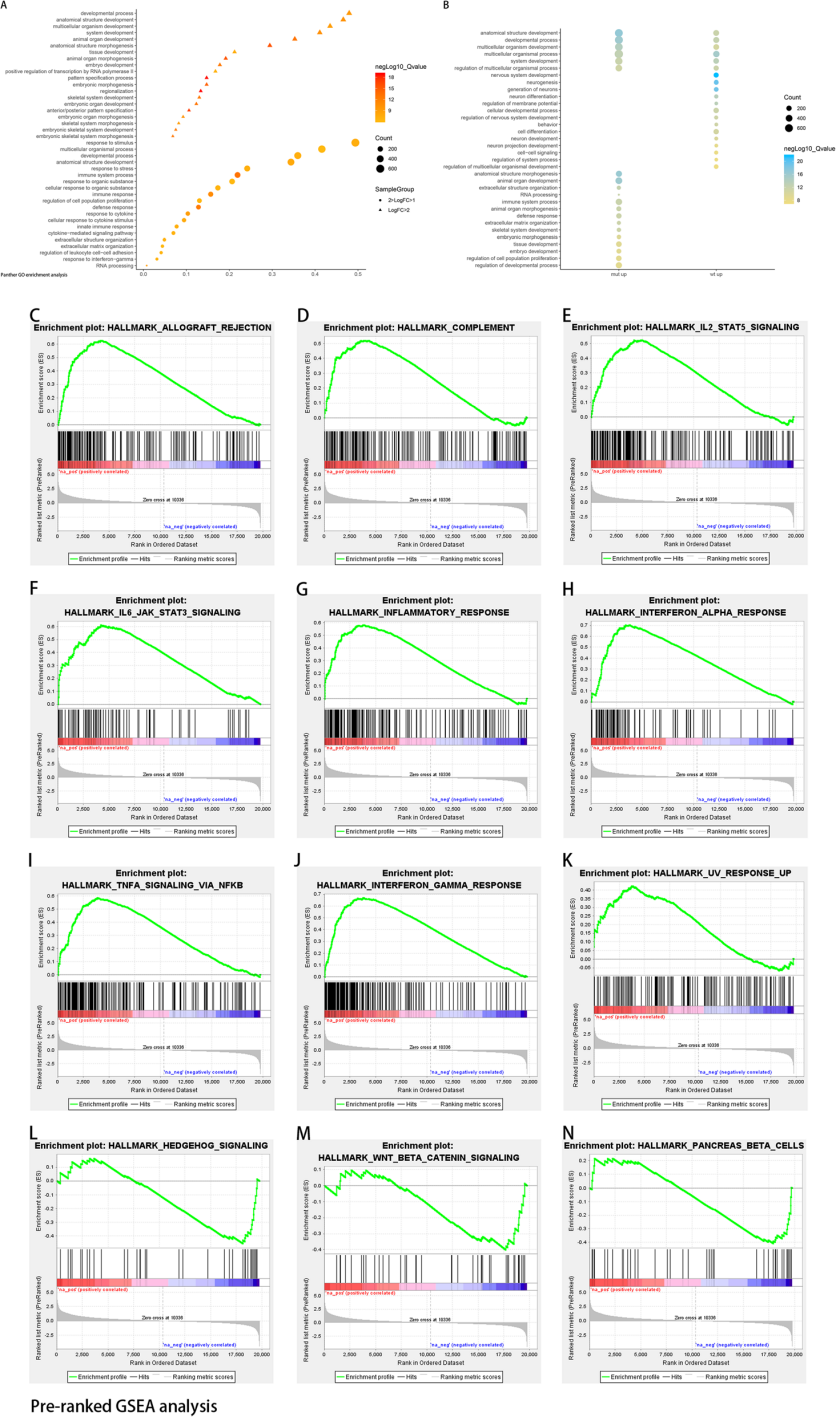
Sectionalized GO analysis and GSEA enrichment. **a**-**b** Gene enrichment analysis was performed using tools available at PANTHER. Babble plot showed enriched processes and pathways in EGFR-MUT LGGs. **c**-**n** Gene Set Enrichment Analysis (GSEA) was performed using genes with FDR < 0.05 and log2FC greater or less than 1.0

To meticulously analyzed the enriched gene sets, sectionalized GO analysis was performed based on the grouping strategy of dividing into Log2FC ≥ 2 set and 2 > Log2FC ≥ 1 set. The results showed that gene sets involved in biological development were principally enriched in the Log2FC ≥ 2 set. While immune response related gene sets were dominantly enriched in the 2 > Log2FC ≥ 1 set (Fig. 2.A-B).

Gene Set Enrichment Analysis (GSEA) was also enrolled to analyze the differential expressed genes set. A pre-ranked gene list was generated based on Log2FC values from the Limma analysis, and the list were then performed GSEA pre-ranked analysis with the hallmark gene sets used as the background. Total 31 gene sets were significantly upregulated in EGFR-MUT cases and 3 gene sets were enriched in EGFR-WT cases at FDR < 25%. Several immune response related gene sets such as interferon-γ response, interferon-α response, allograft rejection, TNFα signaling via NFκB, inflammatory response, IL6-JAK-STAT3 signaling, IL2-STAT5 signaling, complement and UV response were significantly enriched in EGFR-MUT cases. While the gene sets significantly enriched in EGFR-WT cases were hedgehog pathway, pancreas β cell and β-catenin pathway. The results were in consonance with Gene Ontology enrichment analysis. Details were showed in Fig. 2.C-N.

### Enrichment of immune response gene sets correlate with infiltration of specific immune cell types in EGFR mutant LGGs

As immune response related gene sets were characteristically enriched in EGFR-MUT cases, we investigated whether this enrichment trait was correlated with increased tumor infiltrated immune cells (TIIC). The immune cell infiltration levels were compared using data available at TIMER and Wilcoxon test between EGFR-MUT and EGFR-WT subset.

**Fig. 3:**
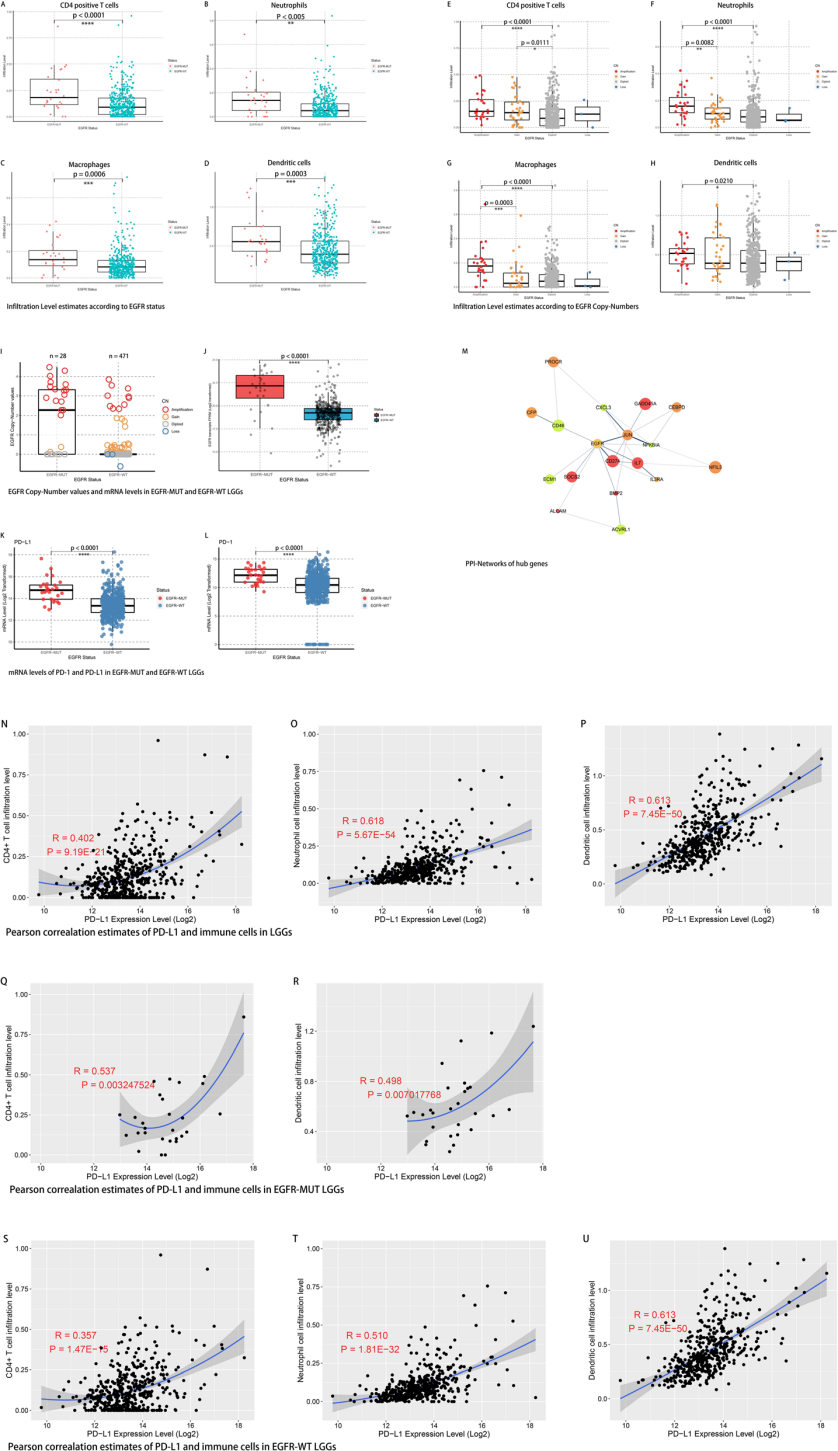
Estimates of tumor infiltrating immune cells along with EGFR, PD-1 and PD-L1 alterations. **a**-**d** Estimates of immune cells infiltration levels according to EGFR mutation. **e**-**h** Estimates of immune cells infiltration levels according to EGFR copy-number alteration. **i**-**j** EGFR copy-number and mRNA level according to EGFR mutant LGGs. **k**-**l** PD-1 and PD-L1 were elevated in EGFR mutant LGGs. **m** The protein–protein interaction (PPI) network of the identified survival associated hub genes. **n**-**p** Pearson correlation analysis of PD-L1 and levels of tumor infiltrating immune cells in whole cohort. **q**-**r** Pearson correlation analysis of PD-L1 and levels of tumor infiltrating immune cells in EGFR-MUT LGGs. **s**-**u** Pearson correlation analysis of PD-L1 and levels of tumor infiltrating immune cells in EGFR-WT LGGs

Consistent with the GO and GSEA results, the present results revealed a higher infiltration level of CD4+ T cells, neutrophils, macrophages, and dendritic cells in EGFR-MUT cases (Fig. 3.A-D). To further explore the relationship between EGFR mutation and TIIC, the infiltration levels were analyzed according to EGFR copy number variations, by using data available at TIMER. Infiltration levels of subsets of CD4+ T cells, neutrophils, macrophages, and dendritic cells were significantly increased with EGFR level amplification cases compared to diploid cases (Fig. 3.E-H). For neutrophils and macrophages subsets, infiltration levels in EGFR amplification (Copy-number > 2) cases were significantly higher than those in EGFR level-gain (2 > Copy-number > 0) cases. Additionally, EGFR copy-number and mRNA level were observed significantly higher in EGFR-MUT LGGs (Fig. 3.I-J). Remarkable correlation of increasing copy-number and mRNA level of EGFR was observed in those EGFR copy-number gained or lost cases (Additional file 8: Fig. S3).

On the other hand, relationships were analyzed between EGFR mutation and genes from enriched immune response pathways. To extracted genes which actively participate in the immune response changes triggered by EGFR mutation, we chose the genes that were significantly associated with clinical outcomes both in EGFR-MUT and EGFR-WT cases (FDR < 0.05). A total of 34 genes met this criterion (Additional file 4: Table S4). Then we unexpectedly found that PD-L1 and PD-1 were elevated in EGFR-MUT LGGs at mRNA level (Fig. 3.K-L). Protein-protein interaction (PPI) network analysis demonstrated that CD274 (PD-L1) was one of the hub genes among this dataset (Fig. 3.M). And a Pearson correlation analysis revealed that PD-L1 expression was correlated with higher TIICs infiltrations in LGGs to a certain extent (Fig. 3.N-U). A hypothesis was consequently emerged that: The increasement of both EGFR copy number and mRNA level might cause a series of biological changes including a PD-L1 elevation and rise the TIICs level, eventually change the immune microenvironment. Similar immune signature was previously reported in EGFR mutation-positive non-small-cell lung cancer (NSCLC) [28–30], in which, elevated PD-L1 in EGFR mutant tumor was consider to be an indicator of better overall response rate to PD-1 inhibitors.

### Increasing infiltration of immune cells correlates with poor overall survival

**Fig. 4:**
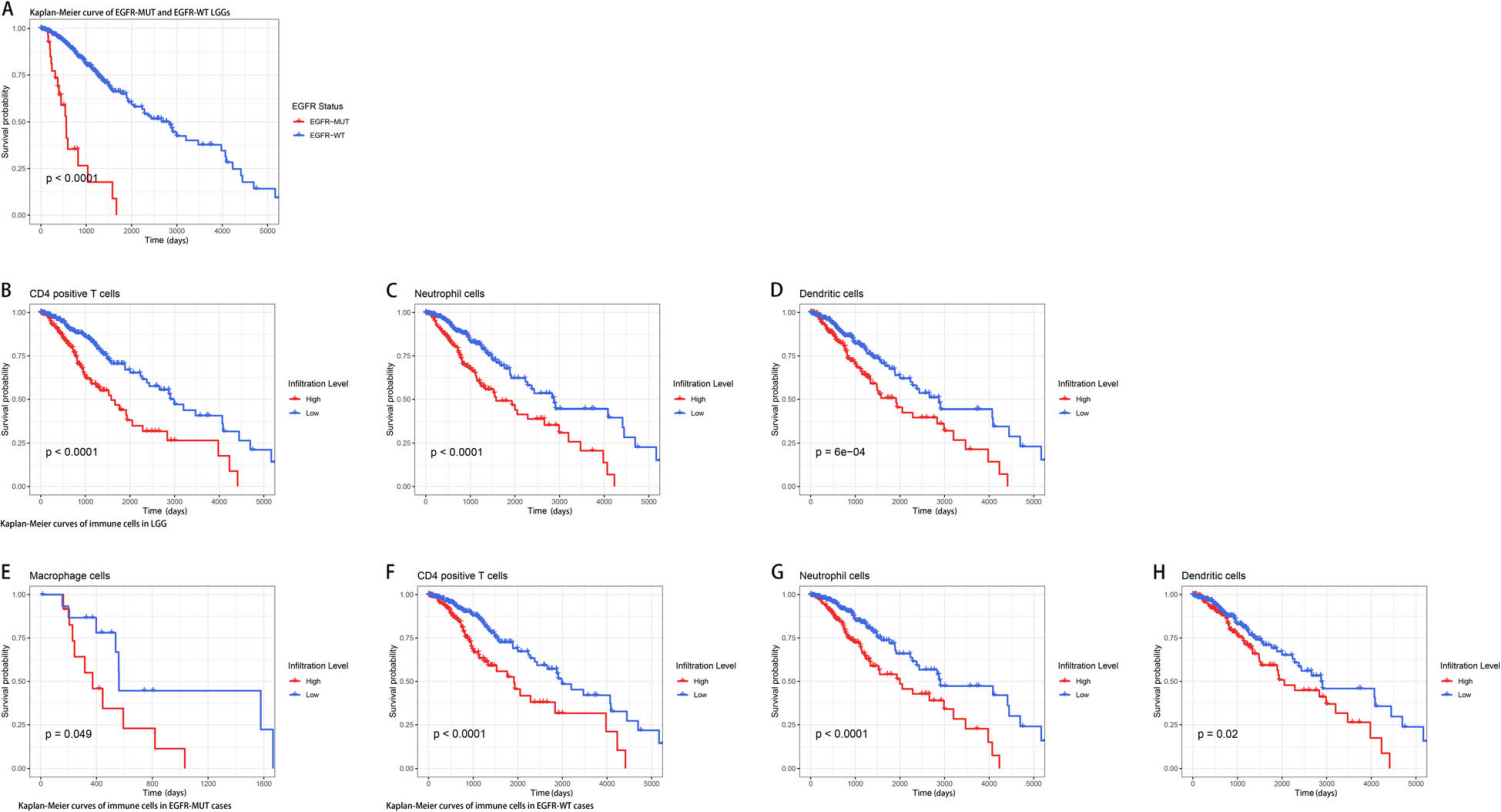
Survival analysis of EGFR mutant LGGs and subsets of tumor infiltrating immune cells in LGGs. **a** EGFR mutation leads to a dismal prognosis. **b**-**d** Kaplan-Meier curves of CD4+ T cells, neutrophils and dendritic cells estimates in LGGs. **e** Kaplan-Meier curve of macrophages estimate in EGFR-MUT cases. **f**-**h** Kaplan-Meier curves of CD4+ T cells, neutrophils and dendritic cells estimates in EGFR-WT cases

High levels of immune cell infiltration were generally considered correlated with better survival in diverse tumors. We inspected whether there was a resembling effect in the survival of LGG patients with or without EGFR mutation. Survival analysis was performed based on the grouping of EGFR mutant and infiltration levels of different types of immune cells respectively. We found a significant difference in the survival of patients with and without EGFR mutation (*p* < 0.0001), displaying an inferior prognosis associated with EGFR mutation. And identified infiltration of CD4+ T cells, neutrophils, macrophages, and dendritic cells possess excellent biomarker potential for monitoring prognosis (Fig. 4.A-D). Therefore, we particularly investigated the effects of infiltration of immune cells on clinical outcomes in all LGG cases enrolled, as well as in EGFR-MUT and EGFR-WT respectively. In the whole cohort, higher CD4+ T cells, neutrophil cells and dendritic cells infiltration levels were correlated with worse survival outcomes. Similar conclusions were also acquired in EGFR-WT cases. As for EGFR-MUT cases, macrophage cells infiltration was observed correlated with poor prognosis (Fig. 4.E-H).

**Fig. 5:**
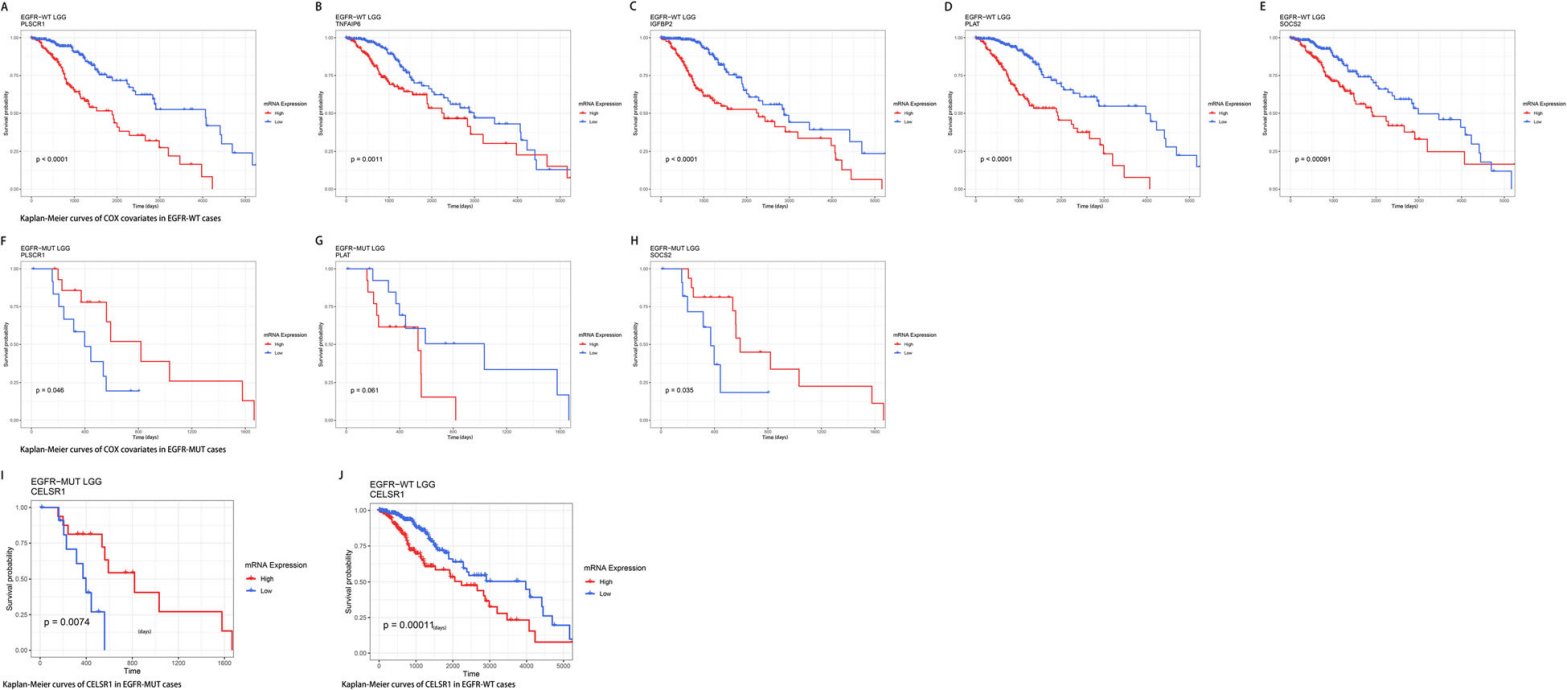
Survival analysis of COX model identified covariates in subsets of LGGs. **a**-**h** Survival analysis of covariates in COX model established on enriched genes and levels of tumor infiltrating immune cells. **i**-**j** Survival analysis of CELSR1 in COX model established on enriched genes and levels of tumor infiltrating immune cells

To further explore the possible explanation of correlation of EGFR mutations and dismal prognosis, we established COX proportional hazards models using genes enriched in EGFR-MUT LGGs or EGFR-WT LGGs and infiltration levels of all types of immune cells (details shown in Additional file 5: Table S5). Four genes, enriched in EGFR-MUT cases: PLSCR1, TNFAIP6, IGFBP2 and PLAT, rose the hazard ratio (HR) in EGFR-WT cases at FDR < 0.05. SOCS2 on the other hand reduced the hazard ratio in EGFR-WT LGGs with a *p* value of 0.027. PLSCR1, a regulator of cell proliferation, maturation, apoptosis and differentiation in leukemia [31]. TNFAIP6 is constitutively expressed in adult CNS and involved in astrocyte-mediated glial formation [32]. IGFBP2 was previously reported to augment the nuclear accumulation of EGFR and to potentiate STAT3 transactivation via nuclear EGFR signaling pathway in glioblastoma [33]. PLAT, also known as tissue-type plasminogen activator (tPA), is thought to regulate vascular-genesis in tumors [34, 35]. And SOCS2, identified as a STAT3 suppressor, acts in a negative feedback loop as regulators of cytokine-triggered cell signaling [36]. Afterwards, we performed log-rank test of these covariates in COX model specifically in EGFR-MUT and EGFR-WT cases. Results showed that higher expression of IGFBP2, PLSCR1 and PLAT is correlated to poor overall survival in EGFR-WT cases, and similar effects were observed in TNFAIP6 or SOCS2 high expression cases at the window of approximately 12 years. However, opposite effects were observed for EGFR-MUT cases, highly expressed SOCS2 or PLSCR1 significantly reduced the survival risks at FDR < 0.05 and associated with better outcomes (Fig. 5.A-H). CELSR1, a core gene from planar cell polarity (PCP) signaling pathway regulating development of the cerebral cortex [37], which was found enriched in EGFR-WT cases and could reduce the hazard ratio (HR = 0.632, *p* value = 0.05) significantly in EGFR-MUT cases. Log-rank test showed that increased expression of CELSR1 was associated with better OS in EGFR-MUT cases, but higher level of CELSR1 indicated worse survival in EGFR-WT cases at the threshold of approximately 14 years (Fig. 5.I-J).

## Discussion

Gliomas were universally regarded as heterogeneous tumor with plentiful genetic alterations. Our study focused on the most frequently altered genes in gliomas. So far TP53 and IDH1 are the most highlighted and studied alteration in LGG, nevertheless EGFR mutation in LGG is a potential underestimated factor on monitoring glioma biological characteristics. According to TCGA data, frequency of EGFR mutation in GBM and LGG is 26.97 and 6.86% respectively. However, recent research has revealed a higher EGFR mutation frequency of 23% in LGGs, attributing to the different population of patients and deeper sequencing depth [38]. It gave us one hint that frequency of EGFR mutation might be notably underestimated, and it also might be the potential explanation of some rapid progressed lower-grade glioma (histopathological diagnosis) which might essentially be higher-grade glioma in molecular level.

**Fig. 6:**
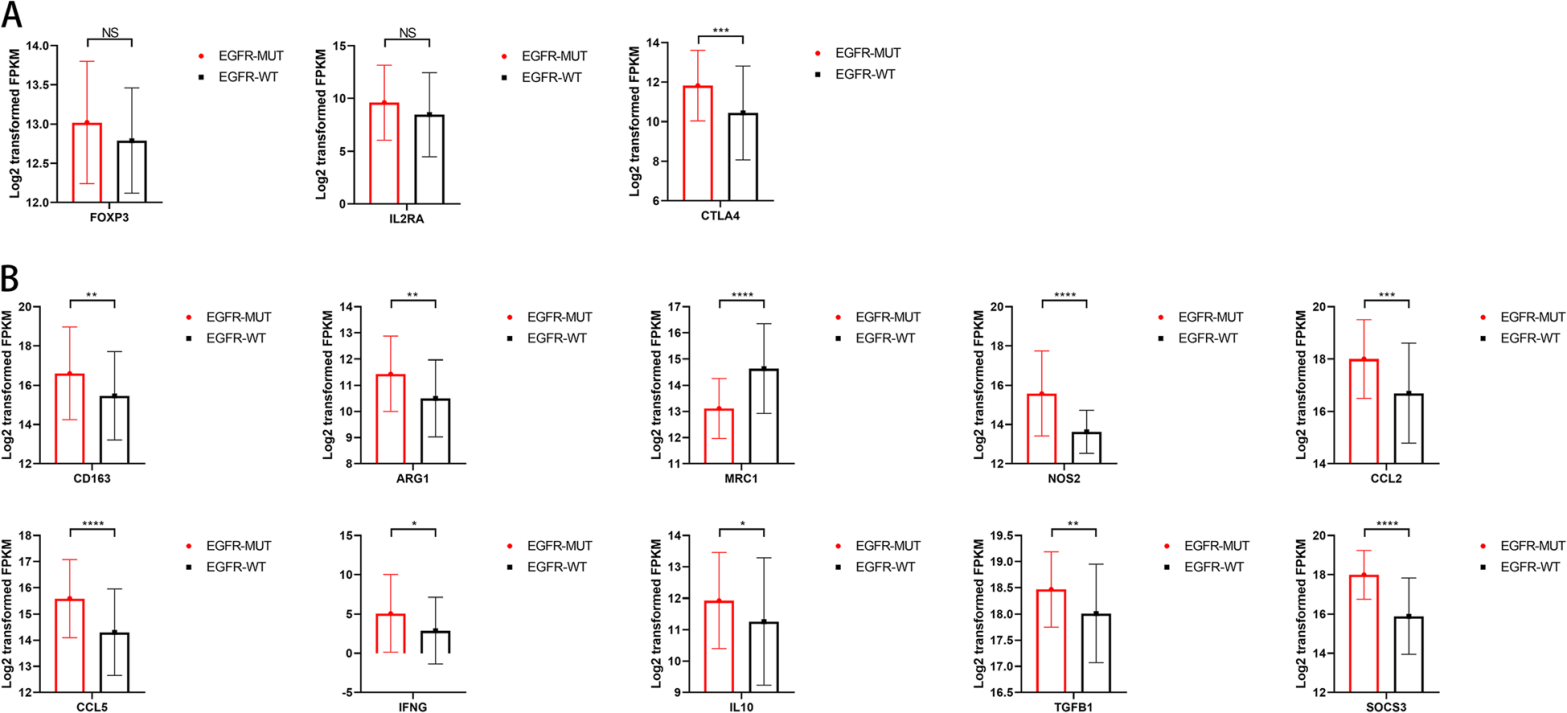
Estimation of marker genes mRNA level of CD4 positive cells and macrophages. **a** mRNA level estimates of potential CD4+ Treg cell markers. **b** Marker genes and cytokines mRNA level estimates of macrophages

EGFR-MUT LGGs were observed to harbor worse overall survival due to the characteristics of amplification of EGFR and elevated level of EGFR mRNA with higher probability, and the following initiation of cascaded downstream effects such as RTK related pathways. GSEA and layered GO analyses revealed that an enrichment of immune response-related gene sets in EGFR mutant cases. Besides, pathways such as epithelial-mesenchymal-transition (EMT), angiogenesis, hypoxia, myogenesis and cholesterol homeostasis etc. were found to more significantly enriched in EGFR-MUT LGGs, indicating the different patterns of histopathology, tumor microenvironment (TME) and metabolism process in EGFR mutant positive and negative LGGs. To better understand of these enrichment results, we therefore enrolled immune infiltration analysis of multiple immune cells and found that CD4+ T cells, neutrophils, macrophages, and dendritic cells were increasingly infiltrated in EGFR-MUT. No significant differences of infiltrated CD8+ T cells and B cells was found between EGFR-MUT and EGFR-WT cases (Additional file 6: Fig. S1). Normally, infiltrating immune cells are considered better survival linked factor in other types of tumor like gastric cancer [39]. High level of tumor infiltrating immune cells in lower-grade glioma were observed to associated with poor clinical outcomes except for macrophages, indicating the possible different population and functions of tumor infiltrated immune cells in glioma. We further investigate the effects of certain infiltration of immune cells in particular EGFR-MUT cases and EGFR-WT cases. The results suggested that macrophages infiltration became a significant (*p* = 0.049) adverse factor of survival particularly in EGFR-MUT LGGs. As for EGFR-WT, the log-rank tests showed a consistency of preceding results that increased infiltration of CD4+ T cells, neutrophils and dendritic cells associated with worse OS. We came out at least two possible hypotheses to explain the impacts of different immune cells on survival. (a) Chronic inflammation is a hallmark of tumor [40]. Instead of acute inflammation that function as infection clearing, heal wounding or maintaining tissue homeostasis, tumor-related inflammation is always chronic and mild. Many factors trigger the inflammatory response in tumors, and activation of oncogenes is one of the crucial factors. Oncogenes like RTKs are often persistently activated in a ligand-independent manner. Emerging literature supports a role of RTKs in inflammation induction [41]. EGFRvIII is the most prevalent ligand-independent phenotype of EGFR alterations, and might causes a tumor-promoting chronic inflammation. (b) The enriched processes in EGFR-MUT cases such as EMT, angiogenesis, myogenesis and cholesterol homeostasis gave us a hint that the TME and metabolism might have changed. First place high mutation burden combined with EMT process might lead to neoantigen genesis and change the presentation processes in TME. Tumor infiltrated immune cells including cytotoxic T lymphocytes, regulatory T cells (Treg) and B cells are quite common in TME. Among them, some are tumor lethal cells and some of them play tumor-promoting roles [41]. For instance, gamma/delta T cells can suppress T and dendritic cell functions [42], Treg cells possesses strong suppressive functions in TME [43]. Since the negative effects of infiltrated CD4+ T cells on survival outcomes, we may hypothesize that immunosuppressive T cells like Treg cells were the major population. Levels of FOXP3 and IL2RA, markers of Treg cells, were not identified express higher in EGFR-MUT samples (Fig. 6.A). However, the key immunosuppressive factor CTLA-4 was confirmed elevated in EGFR-MUT cases, which indicating a possible existence of Treg cells in lower-grade glioma context. Additionally, the prevalent existence of tumor-associated macrophages (TAMs) in gliomas contribute to tumor growth, metastasis and angiogenesis [44]. Several TAMs factors were estimates higher expressed in EGFR-MUT samples (Fig. 6.B). Although the results of CD206(MRC1) and IFN-gamma might lead to the M1 phenotype of macrophage, the even higher level of cytokines like IL-10, TGF-beta, CCL2 and CCL5 and arginase 1(ARG1) in EGFR-MUT samples still be the significant patterns of TAMs, and which indicating more possibly an immunosuppressive population of macrophages. Landscape of cytokine profiling was also generated, and several chemokines that can attract immune cells were identified elevated in EGFR-MUT samples (Additional file 7: Fig. S2). Comprehensively, the alteration of EGFR in LGG might changes the TME and triggers a tumor-associated chronic inflammation, which eventually affect the functions of tumor infiltrated immune cells.

There’s also one exciting clue obtained that PD-1 and PD-L1 were observed elevated in EGFR mutant LGGs and correlated with higher level of infiltrative immune cells. Unlike the strategy of targeted therapy in EGFR mutant NSCLC, neither TKIs nor immune checkpoint inhibitors were widely used in glioma at present. But the pattern of EGFR mutation along with higher expression level of PD-L1 was identified correlate with better response rate of PD-1 inhibitor [28–30], indicating EGFR-mutation might be a novel biomarker monitoring a better outcome of PD-1 inhibitor treatment.

Nonetheless, further particular experiments are needed to identify the detail mechanisms of this pattern. Our results provide some hints for ulterior researches such as focusing on the composition of CD4+ T cells as well as macrophages infiltrated in LGGs.

On the other hand, the COX hazard tests provided puzzled models in a certain extent. One model, generated from enriched genes in EGFR-MUT cases combined with infiltration levels of immune cells, revealed that PLSCR1, TNFAIP6, IGFBP2 and PLAT acted as adverse factors in EGFR-WT cases, and SOCS2 played a protective role in the EGFR-WT cases. While the log-rank test results revealed that high levels of all these five genes, perplexingly including SOCS2, correlated to shorter OS in wild-type cases. Contrary to universal understanding, SOCS2 expression in EGFR-WT cases was associated with worse overall survival. We therefore performed further investigation and found that SOCS2 expression was higher in EGFR mutant LGGs than that in EGFR wild-type LGGs. Since SOCS2 elevation is often considered to be the consequence of STAT3 activation and acts a negative feedback mechanism [33], SOCS2 might highly express in those STAT hyper-activated cases, of which might originally be greater malignant tumor. Interestingly, PLSCR1 and SOCS2 became protective factor on survival with some certain degree in EGFR-MUT cases, PLAT might still act as a threatening factor in EGFR-MUT cases but not significantly (*p* = 0.061). A same intricate conclusion was observed in another COX model established based on the top enriched genes in EGFR-WT cases along with immune cells infiltration estimates. The model suggested that CELSR1 was a protective factor in EGFR-MUT LGGs, and respective log-rank test was in line with the COX expectation. However, when CELSR1 performed with log-rank test in EGFR wild-type LGGs, CELSR1 unexpectedly became a detrimental factor on survival. Eventually, one fact that can’t be denied is that the amount of EGFR mutated cases enrolled were not enough, which could make certain influence on survival analysis results. Enlarged scale of researches were necessary to further confirmed the validities of these varying prognosis indicators in LGG.

**Fig. 7:**
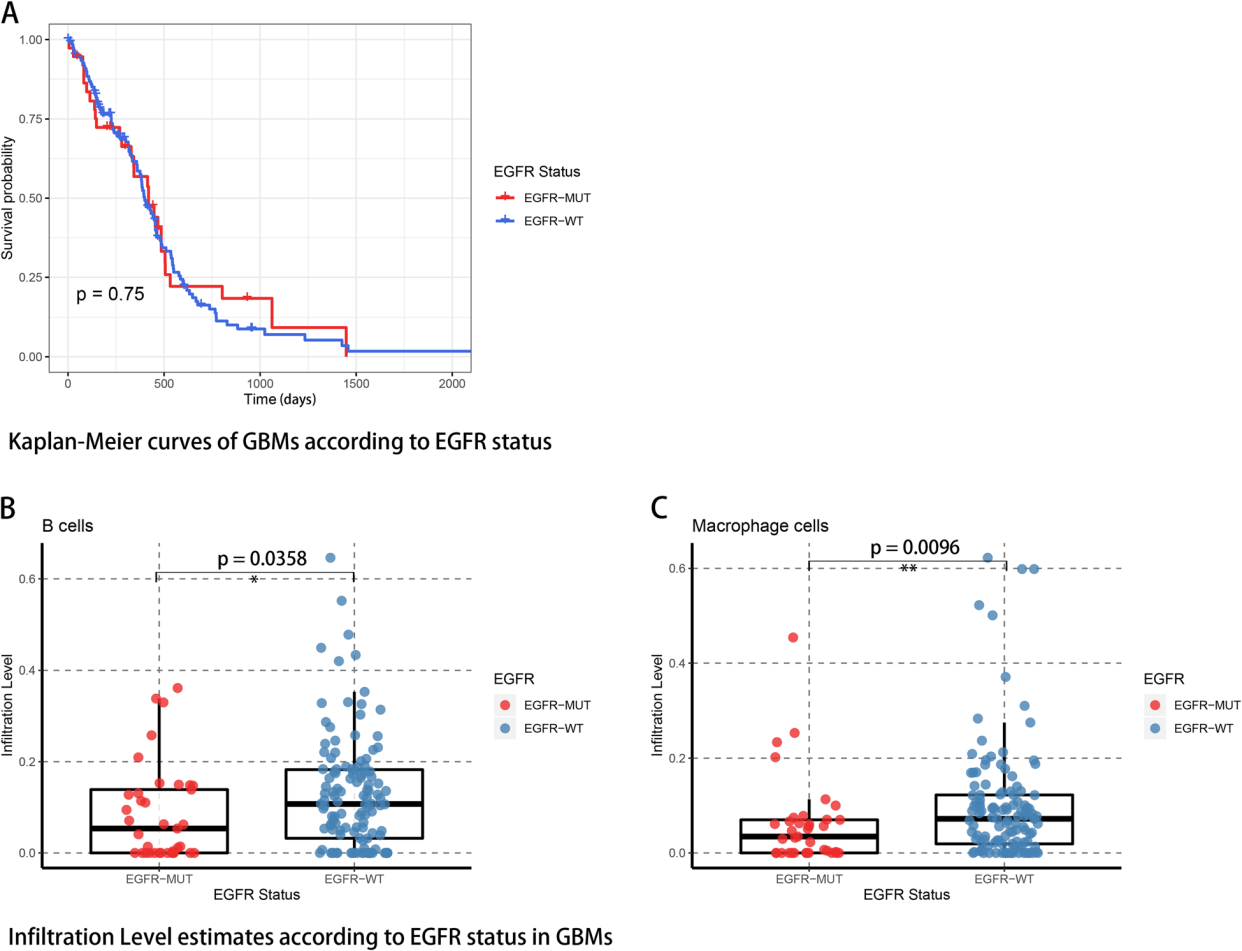
Survival analysis of EGFR mutation in GBM and tumor infiltrating immune cells estimates in EGFR mutant GBMs. **a** EGFR mutation is not an OS predictive factor in GBM. **b**-**c** B cells and macrophages were observed less infiltrated in EGFR-MUT GBMs

Another hallmark for Lower grade glioma is the high rate of recurrence and progression to glioblastoma. We thus performed the similar analysis in this study on GBM, nevertheless, the results didn’t meet our expects. GBM samples were included using the same standard as those cases in LGG, and eventually 160 samples were enrolled within 35 EGFR mutated cases. According to TCGA data, EGFR mutated GBMs showed no significant difference on survival times (Fig. 7). Wilcoxon rank test comparing infiltration levels of immune cells revealed that B cells (*p* = 0.0358) and macrophages (*p* = 0.0096) were less infiltrated in EGFR-MUT GBMs. Disappointingly, the amount of differentially expressed genes were not satisfactory and not able to perform further enrichment analysis. So far, numerous further experimental corroborations were necessary to fully understand the different impacts of EGFR mutation signature on GBM and LGG.

## Conclusions

We described a novel hallmark of EGFR mutation in Lower grade gliomas (WHO grade II-III), and established a connection between noted tumor-related gene phenotype, tumor immune microenvironment and clinical prognosis in LGG. From another point of view, we provide a new scenario on explaining that EGFR mutated LGG cases always harboring worse prognosis, and raise a new potential marker of indicating the prognosis and immune microenvironment status. We also provide a novel indicator of PD-1 inhibitor treatment in glioma, which possesses considerable potential on clinical application.

## Supplementary information

**Additional file 1: Table S1.** VEP prediction. The Variant Effect Predictor (VEP) outcomes of predictions on biological significance of EGFR variants. (CSV 70 kb)

**Additional file 2: Table S2.** Limma analysis of DEGs. The outcomes of analysis of identifying differential expressed genes using Limma package of R. (CSV 195 kb)

**Additional file 3: Table S3.** GO results. The results of Gene Ontology terms. (CSV 23 kb)

**Additional file 4: Table S4.** PPI hub genes. The identified hub genes and the outputs of Log-rank tests. (CSV 166 kb)

**Additional file 5: Table S5.** The COX model. The original parameters and output of COX model established by SPSS version 19. (CSV 2 kb)

**Additional file 6: Figure S1.** Infiltration of CD8+ T cells and B cells. Infiltration estimates of CD8+ T cells and B cells in EGFR-MUT and EGFR-WT cases, no significant differences were found.

**Additional file 7: Figure S2.** Cytokine Profiling heat maps. Chemokines, lymphokines and phenotype markers of macrophages estimations of EGFR-MUT and EGFR-WT cases were revealed using heat maps.

**Additional file 8: Figure S3.** Demonstration of EGFR mRNA level according to copy-number variation. A remarkable positive correlation was found that variation of EGFR mRNA level is synchronous to copy-number variation.

## Data Availability

Raw data of LGG cohort was acquired from TCGA portal (https://portal.gdc.cancer.gov/). Analyzed data is available within supplementary files or from the first author upon reasonable request.

## Abbreviations

CNS: Central nervous system
HGP: Human Genome Project
LGG: Lower-Grade glioma
MAF: Mutation Annotation Format
RTK: Receptor tyrosine kinase
TCGA: The Cancer Genome Atlas
TKI: Tyrosine kinase inhibitor
WHO: World Health Organization
FPKM-UQ: Fragments aligning per kilobase of transcript per million mapped reads normalized to upper quartile
VEP: Variant Effect Predictor
GO: Gene ontology
GSEA: Gene Set Enrichment Analysis
TIMER: Tumor IMmune Estimation Resource
TPM: Transcripts per million reads
PPI: Protein–protein interaction
STRING: Search Tool for the Retrieval of Interacting Genes
EGFR-WT: Wild-type-EGFR
EGFR-MUT: Mutant-EGFR
TIIC: Tumor infiltrated immune cells
NSCLC: Non-small-cell lung cancer
HR: Hazard ratio
tPA: Tissue-type plasminogen activator
PCP: Planar cell polarity
EMT: Epithelial-mesenchymal-transition
TME: Tumor microenvironment
Treg: Regulatory T cells
TAMs: Tumor-associated macrophages
