# Supplementary figures and images for: EGFR mutation: novel prognostic factor associated with immune infiltration in lower-grade glioma; an exploratory study
Source: BMC Cancer. 2019 Dec 4;19:1184. doi: 10.1186/s12885-019-6384-8 (PMC6894128; doi:10.1186/s12885-019-6384-8)

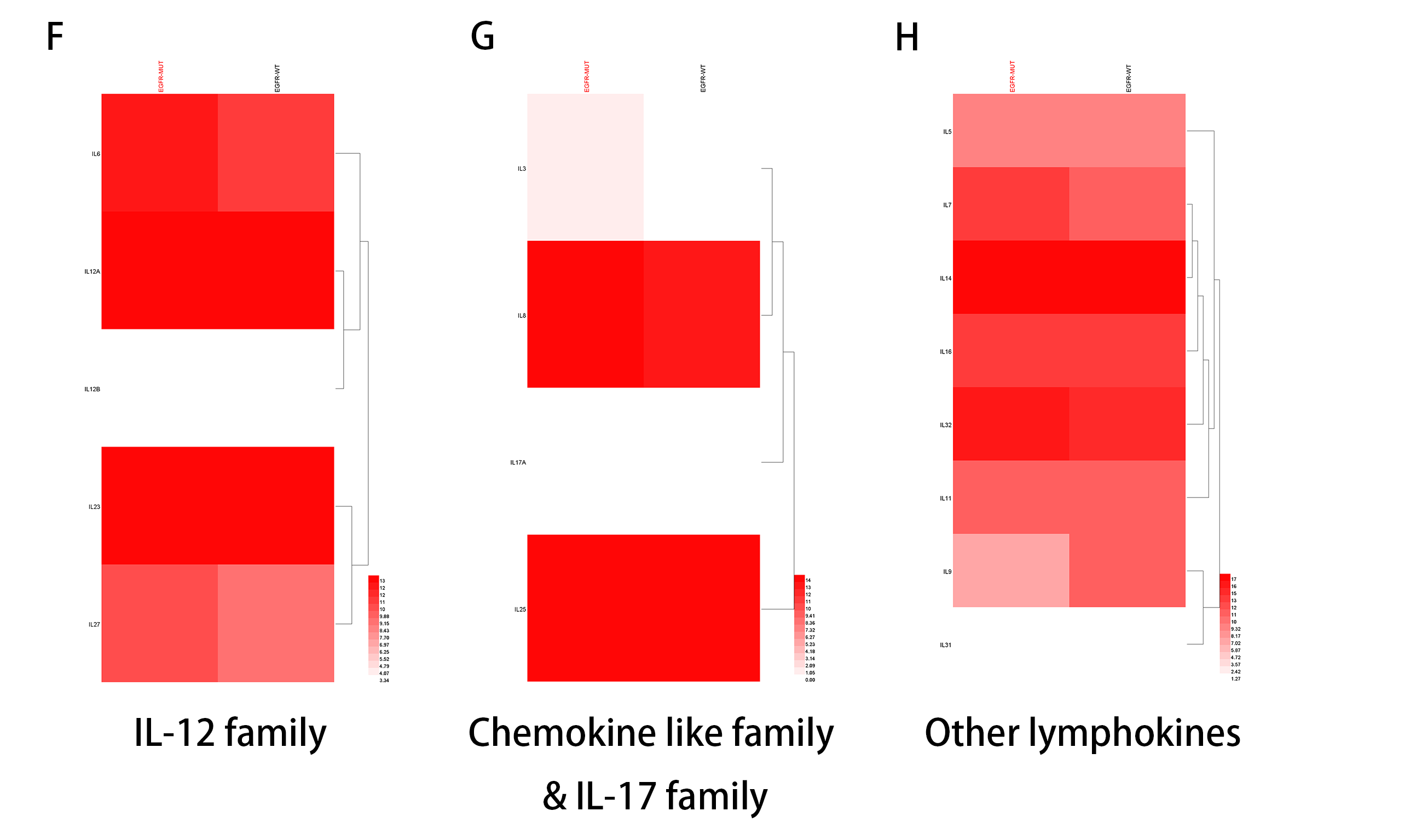

Supplement: Supplementary file 7 — Additional file 7: Figure S2. Cytokine Profiling heat maps. Chemokines, lymphokines and phenotype markers of macrophages estimations of EGFR-MUT and EGFR-WT cases were revealed using heat maps. [file 12885_2019_6384_MOESM7_ESM.zip › Supplementary Fig.2 partCR2.tif]

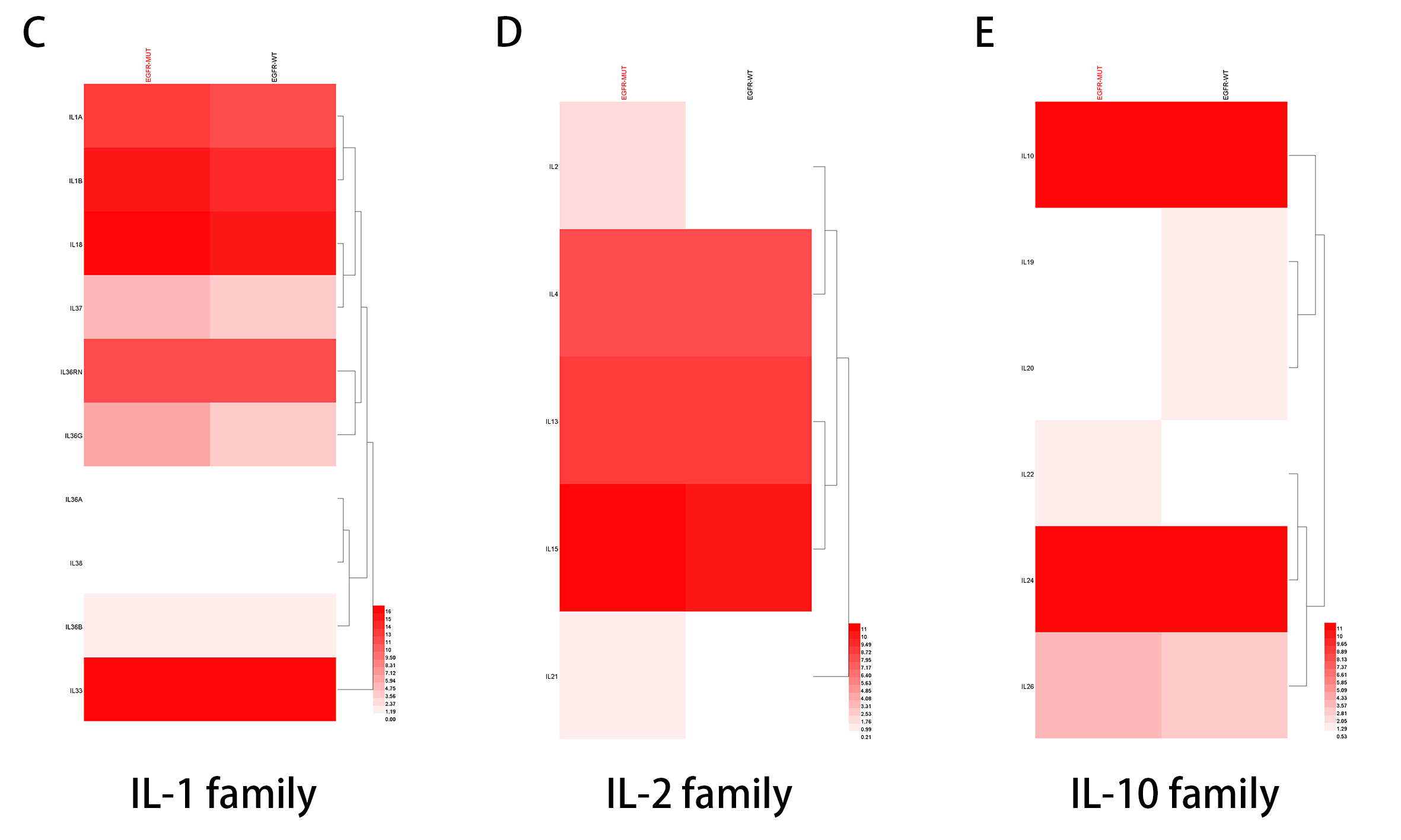

Supplement: Supplementary file 7 — Additional file 7: Figure S2. Cytokine Profiling heat maps. Chemokines, lymphokines and phenotype markers of macrophages estimations of EGFR-MUT and EGFR-WT cases were revealed using heat maps. [file 12885_2019_6384_MOESM7_ESM.zip › Supplementary Fig.2 partBR2.tif]

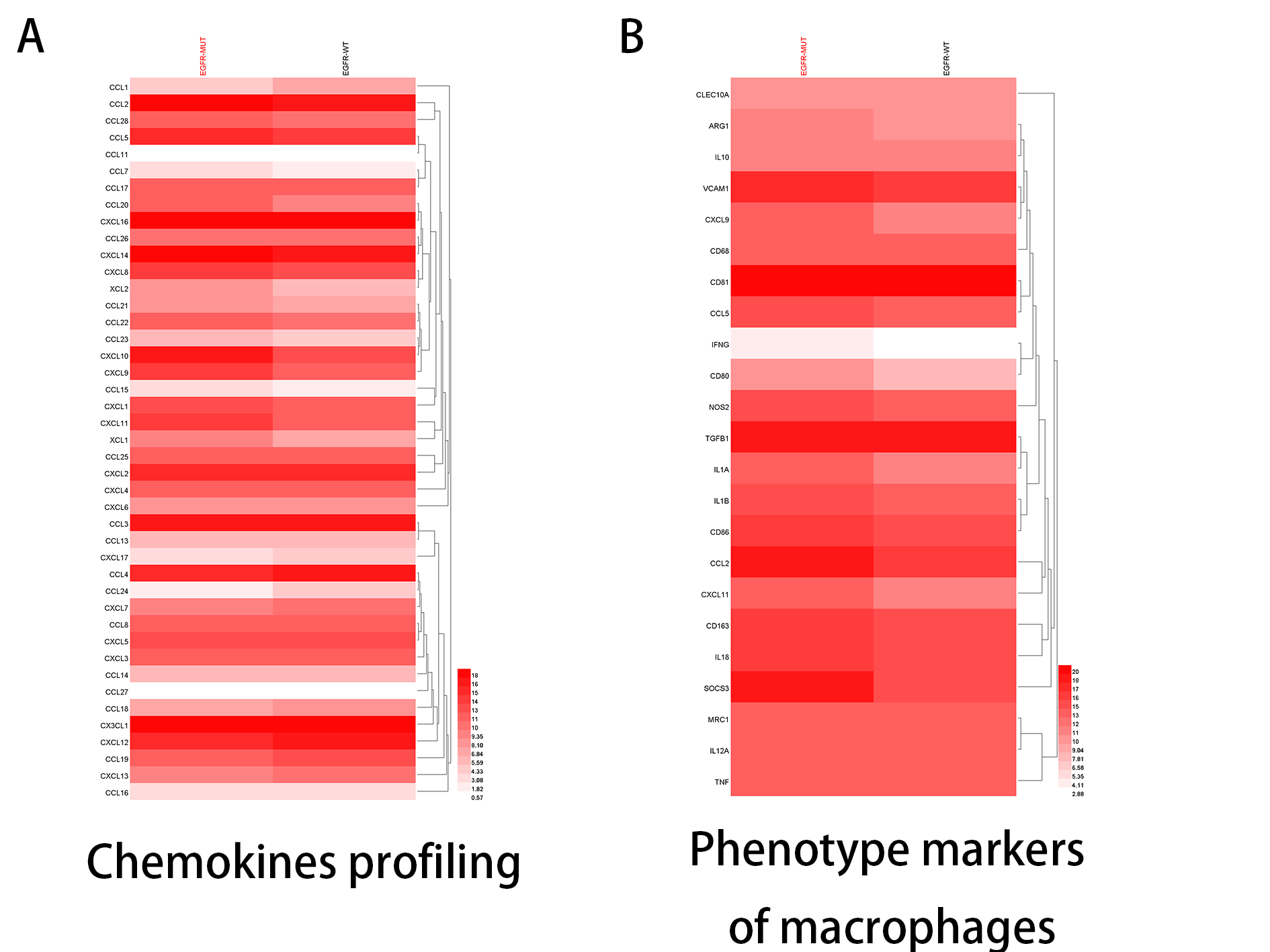

Supplement: Supplementary file 7 — Additional file 7: Figure S2. Cytokine Profiling heat maps. Chemokines, lymphokines and phenotype markers of macrophages estimations of EGFR-MUT and EGFR-WT cases were revealed using heat maps. [file 12885_2019_6384_MOESM7_ESM.zip › Supplementary Fig.2 partAR2.tif]

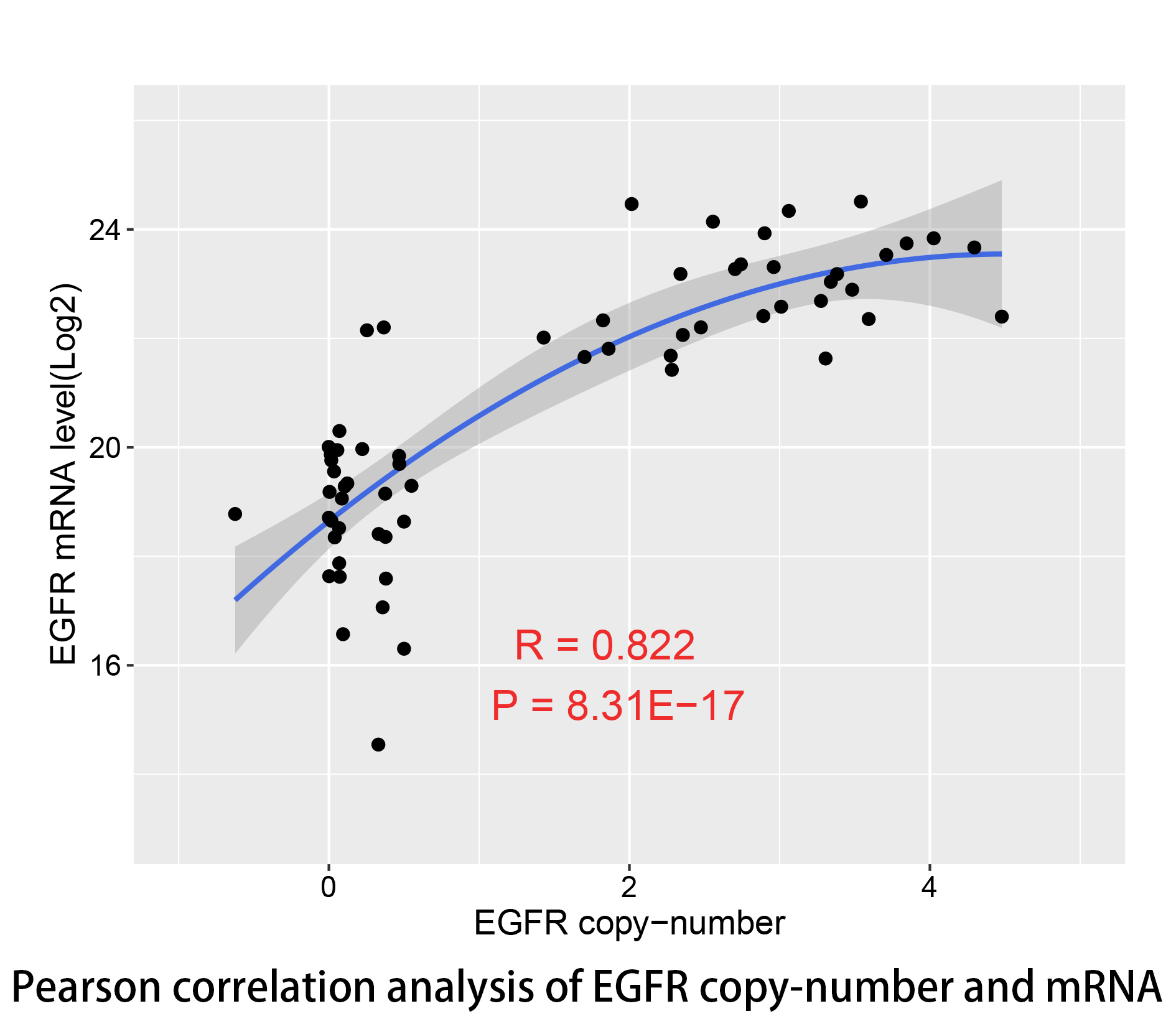

Supplement: Supplementary file 8 — Additional file 8: Figure S3. Demonstration of EGFR mRNA level according to copy-number variation. A remarkable positive correlation was found that variation of EGFR mRNA level is synchronous to copy-number variation. [file 12885_2019_6384_MOESM8_ESM.tif]
